# Lack of Awareness among Future Medical Professionals about the Risk of Consuming Hidden Phosphate-Containing Processed Food and Drinks

**DOI:** 10.1371/journal.pone.0029105

**Published:** 2011-12-29

**Authors:** Yoshiko Shutto, Michiko Shimada, Maiko Kitajima, Hideaki Yamabe, Mohammed S. Razzaque

**Affiliations:** 1 Department of Health Promotion, Hirosaki University Graduate School of Health Sciences, Hirosaki, Japan; 2 Department of Cardiology, Respiratory Medicine and Nephrology, Hirosaki University Graduate School of Medicine, Hirosaki, Japan; 3 Department of Oral Medicine, Infection and Immunity, Harvard School of Dental Medicine, Boston, Massachusetts, United States of America; University of Sao Paulo Medical School, Brazil

## Abstract

Phosphate toxicity is an important determinant of mortality in patients with chronic kidney disease (CKD), particularly those undergoing hemodialysis treatments. CKD patients are advised to take a low phosphate-containing diet, and are additionally prescribed with phosphate-lowering drugs. Since these patients usually seek guidance from their physicians and nurses for their dietary options, we conducted a survey to determine the levels of awareness regarding the high phosphate content in commercially processed food and drinks among medical and nursing students at the Hirosaki University School of Medicine in Japan. For this survey, 190 medical and nursing students (average age 21.7±3 years) were randomly selected, and provided with a list of questions aimed at evaluating their awareness of food and drinks containing artificially added phosphate ingredients. While 98.9% of these students were aware of the presence of sugar in commercially available soda drinks, only 6.9% were aware of the presence of phosphate (phosphoric acid). Similarly, only 11.6% of these students were aware of the presence of phosphate in commercially processed food, such as hamburgers and pizza. Moreover, around two thirds of the surveyed students (67.7%) were unaware of the harmful effects of unrestricted consumption of phosphate-containing food and drinks. About 28% of the surveyed students consume such “fast food” once a week, while 40% drink at least 1∼5 cans of soda drinks/week. After realizing the potential long-term risks of consuming excessive phosphate-containing food and drinks, 40.5% of the survey participants considered reducing their phosphate intake by minimizing the consumption of commercially processed “fast food” items and soda drinks. Moreover, another 48.4% of students showed interest in obtaining more information on the negative health effects of consuming excessive amounts of phosphate. This survey emphasizes the need for educational initiative to raise awareness of the health risks posed by excessive consumption of phosphate additives.

## Introduction

Phosphate is an essential mineral component of the human body, and therefore, its dysregulation can affect the functionality of almost all the organ systems [1], [2], [3], [4], [5], [6], [7], [8], [9]. Phosphate is routinely consumed through food. Both organic and inorganic forms of phosphate are present in regularly consumed foods such as meats, fish, eggs, milk/dairy products, and vegetables. The amount of total phosphate ingestion can be significantly augmented by the consumption of processed food and drinks, as phosphate metabolites are used as additives in these commercially processed food and drinks. In recent years the amount of phosphate intake increased worldwide, especially in countries with a high consumption of processed food. Of particular importance, phosphate, present in the additive is almost entirely absorbed, whereas about 60% is absorbed from naturally available sources.

Recent experimental studies have convincingly demonstrated the risk of increase serum phosphate levels in the development of premature ageing to reno-vascular diseases [8], [9], [10], [11]. Particularly in patients with CKD, hyperphosphatemia is the single most important determinant of mortality [12], [13] perhaps by inducing cardiovascular complications [14], [15], [16]. Since reducing hyperphosphatemia in patients with CKD is one of the main therapeutic priorities, the patients are often advised to consume a low phosphate-containing diet and additionally prescribed with phosphate-lowering drugs. Therefore, healthcare providers, who will be dealing with CKD patients, should be especially aware of the dangers of commercially processed food and drinks, which are often rich in phosphate content.

Developing such awareness is becoming even more important for two main reasons. First, unlike sodium and other food components, phosphate is usually not listed as an ingredient per se, thus making it difficult for patients to avoid phosphate-rich food and drinks. Second, recently, the increased use of phosphate as a preservative has significantly increased in a wide range of drinks and food, complicating the patients' ability to minimize phosphate intake. Healthcare providers including physicians and nurses should provide sufficient guidance and information to their CKD patients to help them reduce their total phosphate intake.

Notably, the number of CKD patients is increasing despite recent progress in biomedical science. In Japan, there are around 13.3 million patients with CKD, while the global prevalence of CKD is estimated to be as high as 500 million [17], [18]. Providing the right guidance by the medical professionals to patients in avoiding phosphate-rich diet could help in decreasing or delaying serious complications of CKD patients with impaired mineral ion metabolism. Therefore, this survey assessed the level of awareness among future medical professionals on artificially added phosphate-containing food and drinks, in a single educational center in Japan.

## Materials and Methods

We randomly selected 190 medical (n = 62) and nursing (n = 128) students who are currently enrolled at the Medical and Nursing Schools in Hirosaki City, Japan. The average age of the medical and nursing students was 24.7±4 and 20.1±1 years, respectively. Out of the total 190 students, 65 were male, 123 were female; two students declined to reveal their gender. Both medical and nursing students completed their 12 years of schooling prior entering to the Medical and Nursing Schools. However, in contrast to the four years of Nursing School, the medical students in Japan need to complete six years of education before dealing with the patients. Japanese medical students are taught Renal Physiology in 3^rd^ year, while, Renal Medicine in 4^th^ year of their course; as for nursing students, Renal Physiology is taught in their 1^st^ year and Renal Medicine in 2^nd^ year of their course, although, the content of the syllabus, especially, Renal Medicine is more detailed for the medical students than the nursing students. All our surveyed medical students were 4^th^ year students, while the nursing students ranged from 2^nd^ to 4^th^ years of their course. All students participated voluntarily on the assurance of anonymity, and none refused to answer the survey questions, although students withhold the answer of a particular question when they were not certain of their response. The questionnaire, assessing each student's levels of awareness of phosphate-containing food and drinks, consisted of seven separate questions (**Table S1**). This survey was conducted during May 2011. In designing the questionnaire, we choose not to examine student's knowledge regarding foods with natural phosphate ingredients; instead, we focused on food and drinks that have phosphate ingredients artificially added as a preservative, such as hamburgers, pizza and soda drinks. Although there are numerous Japanese food items that contain artificially added phosphate ingredients, including commonly consumed instant cup noodles, but to keep this survey simple and uniform, we requested information from the students on hamburgers, pizza or fried chicken, and referred these commonly consumed items as “fast food”. We have clarified the inclusion criteria of “fast food” to the surveyed students beforehand. Study design was approved by the Hirosaki University ethical committee, and all the participants provided informed consent, and participated in this survey voluntarily. The results of the survey were analyzed by Microsoft Excel 2007 and IBM SPSS Statistics 19 software. Mann-Whitney U test was used for comparison between the groups, and p<0.05 was considered as significant.

## Results and Discussion

**Figure 1:**
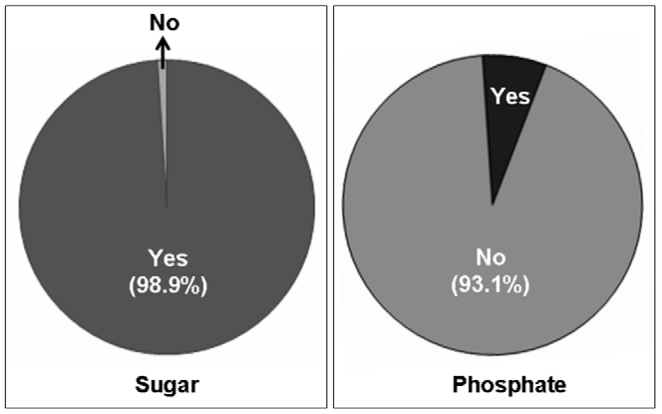
The survey participants were asked whether they were aware of the sugar and phosphate content in commercially available soda drinks. The majority (98.9%) of the participants were aware of the presence of sugar, while only 6.9% were aware of the presence of phosphate (phosphoric acid), showing a huge awareness gap related to phosphate among the participants.

To determine the levels of awareness of future medical professionals regarding the artificially added phosphate-containing food and drinks, we conducted a survey on medical and nursing students currently enrolled at the Medical and Nursing Schools in Hirosaki City, Japan. As patients with CKD are required to maintain a low phosphate-containing diet, healthcare providers should be fully aware of high phosphate-containing “fast food” (hamburgers, pizza or fried chicken) and carbonated soda drinks. Although such awareness is becoming more important, consumers are often uninformed of the phosphate content in such food and drinks or do not truly understand the detrimental effect of a high phosphate-containing diet. Based on this survey, 98.9% of medical and nursing students were aware of the high sugar content in the commercially available soda drinks, while only 6.9% were aware of the presence of phosphate (phosphoric acid) in such drinks. Similarly, only 11.6% of students were aware of the fact that artificially added phosphate, in the form of a preservative, is routinely added in processed food, such as hamburgers and pizza (**Figure 1**). More importantly, 67.7% were not at all aware of the potential harmful consequences of consuming excessive amounts of phosphate for a prolonged period of time.

**Figure 2:**
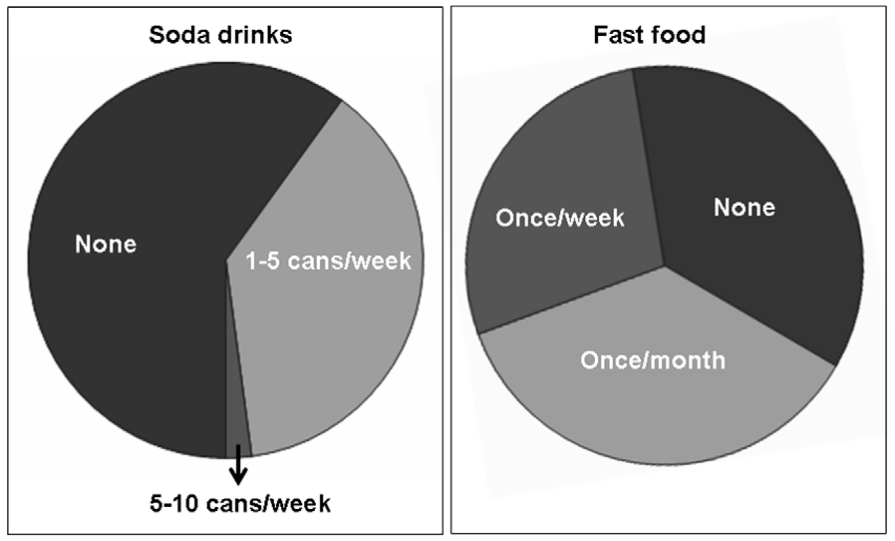
The participants were asked to describe their soda drink and fast food consumption habits. The majority (60%) of participants (average age: 22 years) does not consume soda drinks, while 36% do not eat fast food.

Among the surveyed population, 37.9% of students consumed 1∼5 cans of soda drinks per week, while 2.1% consumed 6∼10 cans of soda drinks per week. Similarly, 28% of students consumed some type of “fast food” (hamburgers, pizza, etc.) once a week, while 36% consumed them once a month (**Figure 2**). We found a clear gender difference in the survey, with a significantly higher proportion of male students consumed soda (p<0.01) and “fast food” (p<0.05); for instance, more than half of the male students consuming 1∼5 cans of soda per week, compared to less than one third of female students. The gender difference found in the consumption of soda drinks and “fast food” is remarkable, given the fact that almost 65% of our surveyed student population is female (**Table S2**).

Notably, the majority (60%) of students did not consume any soda drinks (**Figure 2**). One of the reasons for such a high percentage may be the strong preference for green tea in Japan. One study reported a daily consumption of green tea in 86.7% of Japanese adults (40 to 69 years; n = 13,916) [19]. Such consumption of green tea is not confined only to the elderly individuals, as a study conducted on more than 350 junior high school students in Japan, found that around 40% of students took more than 1 cup of green tea per day during winter [20]. Therefore, provisions of such an alternative healthy drink could minimize the consumption of high sugar- and phosphate-containing soda drinks.

**Figure 3:**
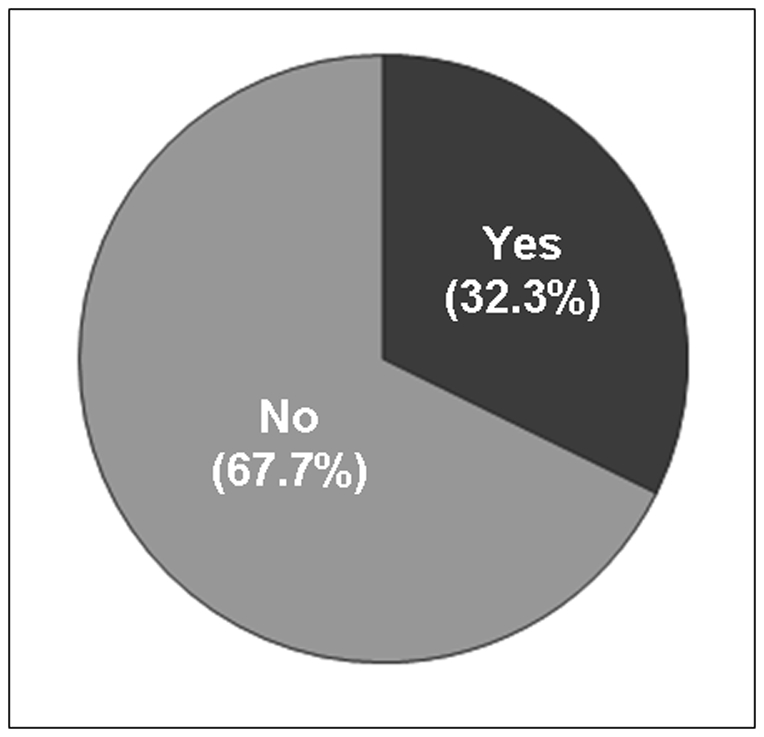
The survey participants were asked whether they were aware of the possible harmful effects of unrestricted consumption of a high phosphate diet for a prolonged period. The majority (67.7%) of participants was unaware of such detrimental effects.

**Figure 4:**
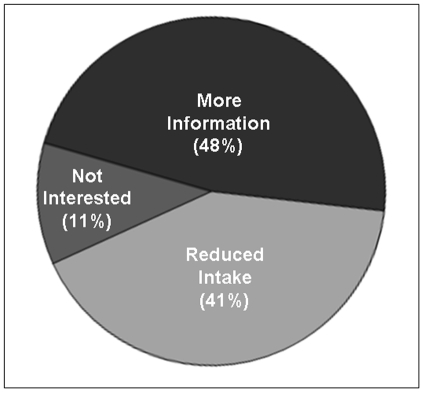
The survey participants were asked whether they were willing to modify their artificially added phosphate-containing diet. Around 48% of the participants wanted to have more phosphate-related information, 41% were willing to reduce their artificial phosphate intake by minimizing consumption of processed food and soda drinks, while the remaining (around 11%) showed no further interest related to phosphate.

In general, the majority (67.7%) of the students in our survey did not appear to appreciate the risk of consuming unrestricted amounts of high phosphate-containing food and drinks (**Figure 3**). Only 6.9% were aware of the fact that most commercially available soda drinks contain phosphate substrate (phosphoric acid). Phosphate-containing soda drinks can influence functionality of different organ systems, as was first reported more than a century ago [21]. Despite such findings, future medical professionals appear to lack awareness regarding the dangers of artificially added phosphate-containing food and drinks. However, 48.4% of them were eager to obtain phosphate-related information, and 40.5% were willing to consider reducing their artificial phosphate intake by minimizing consumption of processed food and soda drinks (**Figure 4**).

There was a clear difference in the approach of male versus female students: around 54% of male students wanted to know more phosphate-related information before taking additional measures. In contrast, about 45% female students were willing to reduce their artificially added phosphate intake by minimizing consumption of processed food and soda drinks. Whether such a flexible approach of female individuals in modifying their dietary habits reflects an overall survival advantage is yet unclear. Notably, the average longevity of Japanese female is 86.4 years, compared to their male counterpart at 79.5 years (source: 2009 statistics compiled and published by the Ministry of Health, Labor and Welfare, Japan).

Even though our survey was conducted on a relatively small population of 190 students in a single educational institute with a higher female ratio (65.4%), the results implicate the necessity of educating future medical professionals about dietary items that contain high levels of artificial phosphate ingredients. Informed healthcare providers will be better equipped to guide their CKD patients to make healthier food choices to reduce complications related to abnormal mineral ion metabolism [22], [23], [24], [25]. Finally, instead of placing nutritionists alone with the task of educating patients to make good food choices, physicians, nutritionists and nurses, in a combined effort, should educate and motivate their patients to consume healthier diet for more effective results.

One important unresolved issue is whether unrestricted phosphate consumption is only detrimental for CKD patients, or whether it has long-term consequences in healthy individuals. In a recent National Health and Nutrition Examination Survey, 11% of elderly people over the age of 65 and without any obvious renal diseases had almost 60% reduced renal function, as compared to non-elderly individuals [26]. As the kidney is a major organ involved in phosphate turnover, consuming excessive phosphate is likely to put additional burden on kidneys in the elderly with compromised renal functions [27]. Moreover, normal kidney function is essential to maintain water, electrolyte and mineral ion balance and to eliminate metabolic waste. Kidneys overburdened with excessive and prolonged consumption of phosphate may become impaired leading to effects on their other essential functions. In fact, studies have convincingly shown that providing high phosphate diet to the healthy individuals could markedly increase fractional urinary excretion of phosphate (FEPi) to maintain homeostatic balance. For instance, compared to the 18.6±5 FEPi for the healthy individuals who consumed 1500 g/day of dietary phosphate, a 30.4±10 FEPi was detected in the same individuals when provided with 2300 g/day dietary phosphate, clearly showing additional workloads by the kidneys to maintain phosphate balance following higher consumption of phosphate [28]. In extreme situations, irreversible renal failure may result in the absence of therapeutic intervention, as reported in a 4-year-old chronically constipated girl with normal renal function who was rectally administered hypertonic phosphate solution that resulted in phosphate toxicity (23 mg/dL) with breathing difficulties and a depressed level of consciousness, even without the presence of predisposing risk factors [29].

Finally, the results of the survey highlight two important points: medical and nursing students, future medical professionals, who will soon assume the role of patient management: **1**) are insufficiently aware of the risks related to prolonged high amount of phosphate intake and **2**) are insufficiently aware of the food and drinks that contain artificially added phosphate ingredients. This survey on future medical professionals exposed the need for an educational initiative to raise awareness of risk posed by dietary items with hidden phosphate ingredients.

## Supporting Information

Table S1 List of questioner.
(DOC)

Table S2 **The survey results compiled separately for medical and nursing students.**
(DOC)
