# Supplementary material for: Lack of Awareness among Future Medical Professionals about the Risk of Consuming Hidden Phosphate-Containing Processed Food and Drinks
Source: PLoS One. 2011 Dec 29;6(12):e29105. doi: 10.1371/journal.pone.0029105 (PMC3248402; doi:10.1371/journal.pone.0029105)
Supplement: Table S1 — List of questioner. (DOC) [file pone.0029105.s001.doc]

**Table S1**

**List of questioner**

1. Do you know carbonated Soda drinks contain high levels of sugar? **Yes / No**
2. Do you know carbonated Soda drinks contain high levels of phosphate? **Yes / No**
3. Do you know “fast foods” contain high levels of phosphate? **Yes / No**
4. Do you know consuming too much phosphate may be harmful for the body? **Yes / No**
5. How many cans of carbonated Soda do you drink a week?

**1-** None,

**2-** 1-5,

**3-** 6-10,

**4**- >10,

1. How often do you eat “fast food”?

**1**- Do not eat,

**2**- Once a week,

**3**- Once a month,

**4**- Almost every day,

1. After knowing that too much phosphate consumption might be harmful, what do you think you should do?

**1-** I want to get more information about phosphate.

**2-** I want to reduce consumption of phosphate-containing food/drink.

**3-** I have no further interest on phosphate-related issues.

* Fast food in this survey is restricted to the commercially available hamburgers, pizza or fried chicken

** Soda in this survey is restricted to the commercially available carbonated drinks
