# Supplementary material for: Lack of Awareness among Future Medical Professionals about the Risk of Consuming Hidden Phosphate-Containing Processed Food and Drinks
Source: PLoS One. 2011 Dec 29;6(12):e29105. doi: 10.1371/journal.pone.0029105 (PMC3248402; doi:10.1371/journal.pone.0029105)
Supplement: Table S2 — The survey results compiled separately for medical and nursing students. (DOC) [file pone.0029105.s002.doc]

**Table S2**

The survey results compiled separately for medical and nursing students.

|  | **n=190** | **Medical students n=62** | **Nursing students n=128** |
| --- | --- | --- | --- |
| **Age** | 21.7±3.5 | 24.7±4.6 | 20.1±1 |
| **Gender** | n=188 (2 did not responded) |  |  |
| **Male** | 65 (34.6%) | 49 (81.7%) | 16 (12.5%) |
| **Female** | 123 (65.4%) | 11 (18.3%) | 112 (87.5%) |
| **Q1** | n=190 |  |  |
| **Yes** | 188 (98.9%) | 62 (100.0%) | 126 (98.4%) |
| **No** | 2 (1.1%) | 0 (0.0%) | 2 (1.6%) |
| **Q2** | n=189 |  |  |
| **Yes** | 13 (6.9%) | 5 (8.0%) | 8( 6.3%) |
| **No** | 176 (93.1%) | 57 (91.9%) | 119 (93.7%) |
| **Q3** | n=189 |  |  |
| **Yes** | 22 (11.6%) | 7 (11.3%) | 15( 11.8%) |
| **No** | 167 (88.4%) | 55 (88.7%) | 112 (88.2%) |
| **Q4** | n=189 |  |  |
| **Yes** | 61 (32.3%) | 24 (39.3%) | 37 (28.9%) |
| **No** | 128 (67.7%) | 37 (60.7%) | 91 (71.1%) |
|  |  |  |  |
| **Q5** | n=190 |  |  |
| **1** | 114 (60.0%) | 34 (54.8%) | 80 (62.5%) |
| **2** | 72 (37.9%) | 25 (40.3%) | 47 (36.7%) |
| **3** | 4 (2.1%) | 3 (4.8%) | 1 (0.8%) |
| **Q6** | n=189 |  |  |
| **1** | 68 (36.0%) | 19 (31.1%) | 49 (38.3%) |
| **2** | 53 (28.0%) | 26 (42.6%) | 27 (21.1%) |
| **3** | 68 (36.0%) | 16 (26.2%) | 52 (40.6%) |
| **Q7** | n=190 |  |  |
| **1** | 92 (48.4%) | 33 (53.2%) | 59 (46.1%) |
| **2** | 77(40.5%) | 17(27.4%) | 60(46.9%) |
| **3** | 21 (11.1%) | 12 (19.4%) | 9 (7.0%) |
